# Residual Lung Abnormalities after COVID-19 Hospitalization: Interim Analysis of the UKILD Post–COVID-19 Study

**DOI:** 10.1164/rccm.202203-0564OC

**Published:** 2022-12-01

**Authors:** Iain Stewart, Joseph Jacob, Peter M. George, Philip L. Molyneaux, Joanna C. Porter, Richard J. Allen, Shahab Aslani, J. Kenneth Baillie, Shaney L. Barratt, Paul Beirne, Stephen M. Bianchi, John F. Blaikley, James D. Chalmers, Rachel C. Chambers, Nazia Chadhuri, Christopher Coleman, Guilhem Collier, Emma K. Denneny, Annemarie Docherty, Omer Elneima, Rachael A. Evans, Laura Fabbri, Michael A. Gibbons, Fergus V. Gleeson, Bibek Gooptu, Neil J. Greening, Beatriz Guillen Guio, Ian P. Hall, Neil A. Hanley, Victoria Harris, Ewen M. Harrison, Melissa Heightman, Toby E. Hillman, Alex Horsley, Linzy Houchen-Wolloff, Ian Jarrold, Simon R. Johnson, Mark G. Jones, Fasihul Khan, Rod Lawson, Olivia Leavy, Nazir Lone, Michael Marks, Hamish McAuley, Puja Mehta, Dhruv Parekh, Karen Piper Hanley, Manuela Platé, John Pearl, Krisnah Poinasamy, Jennifer K. Quint, Betty Raman, Matthew Richardson, Pilar Rivera-Ortega, Laura Saunders, Ruth Saunders, Malcolm G. Semple, Marco Sereno, Aarti Shikotra, A. John Simpson, Amisha Singapuri, David J. F. Smith, Mark Spears, Lisa G. Spencer, Stefan Stanel, David R. Thickett, A. A. Roger Thompson, Mathew Thorpe, Simon L. F. Walsh, Samantha Walker, Nicholas David Weatherley, Mark E. Weeks, Jim M. Wild, Dan G. Wootton, Chris E. Brightling, Ling-Pei Ho, Louise V. Wain, Gisli R. Jenkins

**Affiliations:** ^1^ National Heart & Lung Institute, Imperial College London, London, United Kingdom;; ^2^ Centre for Medical Imaging Computing,; ^13^ Respiratory Medicine, and; ^4^ University College London, London, United Kingdom;; ^3^ Royal Brompton and Harefield Clinical Group, Guy’s and St. Thomas’ NHS Foundation Trust, London, United Kingdom;; ^5^ Department of Health Sciences, University of Leicester, Leicester, United Kingdom;; ^6^ Leicester NIHR Biomedical Research Centre, Leicester, United Kingdom;; ^21^ Usher Institute and; ^7^ University of Edinburgh, Edinburgh, United Kingdom;; ^8^ North Bristol NHS Trust, Bristol, United Kingdom;; ^9^ Leeds Teaching Hospitals NHS Foundation Trust, Leeds, United Kingdom;; ^10^ Sheffield Teaching Hospitals NHS Foundation Trust, Sheffield, United Kingdom;; ^11^ University of Manchester, Manchester, United Kingdom;; ^12^ Ninewells Hospital and Medical School, Dundee, United Kingdom;; ^14^ University of Nottingham, Nottingham, United Kingdom;; ^15^ University of Sheffield, Sheffield, United Kingdom;; ^16^ Royal Devon and Exeter NHS Foundation Trust, Exeter, United Kingdom;; ^17^ Oxford University Hospitals NHS Foundation Trust, Oxford, United Kingdom;; ^18^ University College London Hospital, London, United Kingdom;; ^19^ Asthma + Lung UK, London, United Kingdom;; ^20^ Faculty of Medicine, University of Southampton, Southampton, United Kingdom;; ^22^ University of Birmingham, Brimingham, United Kingdom;; ^23^ Manchester University NHS Foundation Trust, Manchester, United Kingdom;; ^24^ University of Oxford, Oxford, United Kingdom;; ^25^ University of Liverpool, Liverpool, United Kingdom;; ^26^ Newcastle University, Newcastle, United Kingdom;; ^27^ Perth Royal Infirmary, NHS Tayside, Perth, United Kingdom; and; ^28^ Liverpool University Hospitals NHS Foundation Trust, Liverpool, United Kingdom

**Keywords:** COVID-19, hospitalization, HRCT, lung damage, lung abnormalities

## Abstract

**Rationale:**

Shared symptoms and genetic architecture between coronavirus disease (COVID-19) and lung fibrosis suggest severe acute respiratory syndrome coronavirus 2 (SARS-CoV-2) infection may lead to progressive lung damage.

**Objectives:**

The UK Interstitial Lung Disease Consortium (UKILD) post–COVID-19 study interim analysis was planned to estimate the prevalence of residual lung abnormalities in people hospitalized with COVID-19 on the basis of risk strata.

**Methods:**

The PHOSP–COVID-19 (Post-Hospitalization COVID-19) study was used to capture routine and research follow-up within 240 days from discharge. Thoracic computed tomography linked by PHOSP–COVID-19 identifiers was scored for the percentage of residual lung abnormalities (ground-glass opacities and reticulations). Risk factors in linked computed tomography were estimated with Bayesian binomial regression, and risk strata were generated. Numbers within strata were used to estimate posthospitalization prevalence using Bayesian binomial distributions. Sensitivity analysis was restricted to participants with protocol-driven research follow-up.

**Measurements and Main Results:**

The interim cohort comprised 3,700 people. Of 209 subjects with linked computed tomography (median, 119 d; interquartile range, 83–155), 166 people (79.4%) had more than 10% involvement of residual lung abnormalities. Risk factors included abnormal chest X-ray (risk ratio [RR], 1.21; 95% credible interval [CrI], 1.05–1.40), percent predicted Dl_CO_ less than 80% (RR, 1.25; 95% CrI, 1.00–1.56), and severe admission requiring ventilation support (RR, 1.27; 95% CrI, 1.07–1.55). In the remaining 3,491 people, moderate to very high risk of residual lung abnormalities was classified at 7.8%, and posthospitalization prevalence was estimated at 8.5% (95% CrI, 7.6–9.5), rising to 11.7% (95% CrI, 10.3–13.1) in the sensitivity analysis.

**Conclusions:**

Residual lung abnormalities were estimated in up to 11% of people discharged after COVID-19–related hospitalization. Health services should monitor at-risk individuals to elucidate long-term functional implications.

**At a Glance Commentary**

**Scientific Knowledge on the Subject**

Current studies highlight persistent breathlessness and radiological patterns suggestive of lung fibrosis, as well as shared genetic architecture with idiopathic pulmonary fibrosis, in people discharged after severe coronavirus disease (COVID-19) hospitalization. Survivors of COVID-19 may develop parenchymal abnormalities consistent with lung fibrosis.

**What This Study Adds to the Field**

This study assesses the risk factors for residual lung abnormalities, provides evidence of persistent abnormalities within 1 year of discharge from over 200 computed tomography scans, and estimates the prevalence of lung abnormalities after discharge to be up to 11% in a broad range of COVID-19 severity. The findings emphasize the importance for health services to undertake active radiological and physiological monitoring to assess progression or resolution over time.

Long-term symptoms of coronavirus disease (COVID-19) have been widely reported and can have a severe impact on quality of life, frequently characterized by chronic breathlessness (1–3). Postmortem studies on patients with COVID-19 have highlighted diffuse parenchymal alterations, including alveolar damage, exudation, and the development of pulmonary fibrosis, which may explain chronic respiratory symptoms in survivors (4–6).

A number of studies have identified similarities between severe COVID-19 and idiopathic pulmonary fibrosis, an archetypal interstitial lung disease (ILD). These include shared genetic etiology (7, 8), circulating biomarkers (9, 10), similarities in pulmonary function, and radiological features (11). Viral injury may promote lung fibrosis, and chronic viral infection has been shown to be associated with developing idiopathic pulmonary fibrosis (12). Consequently, survivors of COVID-19 may develop parenchymal abnormalities consistent with ILD, including radiological patterns of ground-glass opacities and reticulations.

To understand the potential risk of COVID-19 leading to the development of longer-term ILD and fibrosis, the UK Interstitial Lung Disease Consortium (UKILD) post–COVID-19 study aims to investigate the risk factors and nature of long-term lung damage from COVID-19 in a longitudinal observational study. To support clinical and research management, this planned interim analysis of the UKILD post–COVID-19 study addresses the extent of residual lung abnormalities after hospitalization after completion of an early follow-up visit of the prospective PHOSP–Covid-19 (Post-Hospitalization COVID-19) study (13).

Some of the results of these studies have been previously reported in the form of a preprint (medRxiv, 16 March 2022; https://www.medrxiv.org/content/10.1101/2022.03.10.22272081v2).

## Methods

### Participants

This interim analysis was restricted to participants of the PHOSP–COVID-19 study, a prospective longitudinal cohort study of adults discharged from National Health Service hospitals across the United Kingdom after admission for confirmed or clinically diagnosed COVID-19, previously described in detail (14).

Individuals withdrawing consent from PHOSP–COVID-19 were excluded. Individuals being managed for an *a priori* diagnosed ILD or pulmonary fibrosis, as recorded by site teams using hospital notes, were identified by hand searches of comorbidities and subsequently excluded.

### Interim Study Design

Interim participants were discharged by the end of March 2021, representing wave one of the pandemic; interim data were collected up to October 2021 and were restricted to within 240 days of discharge. Analyses were performed with data recorded through routine follow-up (PHOSP–COVID-19 Tier 1) and those with completed early research follow-up visits (PHOSP–COVID-19 Tier 2). Clinically indicated thoracic computed tomography (CT) scans were identified through the PHOSP–COVID-19 study via linkage to a radiological database; linked CT scans were requested at clinical discretion. The presence of residual lung abnormalities on volumetric CTs was scored on a lobar basis; the percentage involvement of ground-glass opacities, reticulations, or the sum of involvement was averaged across lobes to quantify residual abnormality (15). The primary outcome was visually scored residual abnormalities greater than 10% lung involvement on CT (15).

Risk factors implicated in worse outcomes after COVID-19 hospitalization of individuals with ILD were described (16). These included sex, age, ethnicity, body mass index, and IMD (Index of Multiple Deprivation). A modified WHO (World Health Organization) clinical progression scale was used to define the severity of admission: *1*) no supplemental oxygen; *2*) supplemental oxygen only; *3*) continuous positive airway pressure (CPAP); and *4*) invasive mechanical ventilation (IMV) or extracorporeal membrane oxygenation (ECMO). Abnormal chest X-ray (CXR) reports were classified at follow-up, defined as “suggestive of lung fibrosis,” “extensive, persistent changes greater than one-third of lung involvement,” and “indeterminate,” compared with “other” or “normal.” Breathless and cough symptoms were recorded at follow-up with the patient symptom questionnaire developed for the PHOSP–COVID-19 study (14). Percent predicted values for FVC (ppFVC) and Dl_CO_ (ppDl_CO_) were obtained at follow-up visits and calculated using global lung initiative reference equations.

### Statistical Analysis

Risk factor data were presented descriptively overall, according to PHOSP–COVID-19 tier, and within the sample of linked and scored CTs. Chi-square tests were performed on nonmissing categories. Residual abnormalities on paired CT scans were tested with paired *t* test; changes in scored residual lung abnormalities over time were estimated using linear mixed effect models, with random effects of timing at the level of the individual, adjusted for sex and IMD. A random sample of 70 CT scans was tested for interrater agreement by Cohen’s kappa (κ), with a second radiologist blinded to scores.

Univariate relative risk ratios of threshold greater than 10% residual abnormalities and difference in involvement on CT were modeled with dichotomized exposure variables. Bayesian binomial and linear associations were estimated using 12,500 Markov Chain Monte Carlo (MCMC) iterations, including a burn-in of 2,500 and 10,000 subsequent simulations using random-walk Metropolis-Hastings sampling. Noninformative, flat priors were selected, and estimates were reported with a 95% CrI. Linear associations were additionally adjusted for demographics of sex and IMD.

Clinical risk factors with consistent significant effects were selected to develop risk strata of suspected residual lung abnormalities after COVID-19 hospitalization. For the indexing of risk strata, missing data on clinical indicators were imputed to the reference (lowest risk) category. The percentage of participants within moderate- to very-high–risk strata and no CT scored were defined as at-risk. Hospital admissions were compared between at-risk groups using chi-square, and 15 index admission variables were selected from 61 by least absolute shrinkage and selection operator.

Bayesian inference with a binomial distribution of at-risk cases and noncases (17) was used to estimate the prevalence of suspected residual lung abnormalities after COVID-19 hospitalization within 240 days of discharge, reported with a 95% CrI. MCMC simulations were run as described above. Noninformative, uniform β priors were used and compared in sensitivity analyses with uniform Jeffrey’s priors, as well as skeptical and power priors informed by published population studies of ILD (18, 19). Sensitivity analyses were performed in PHOSP–COVID-19 Tier 2 research follow-up participants in which data completeness was greater. Analyses were performed in Stata SE16.0 within the Scottish National Safe Haven Trusted Research Environment.

## Results

### Cohort Demographics and Patterns of Lung Damage

**Figure 1.**
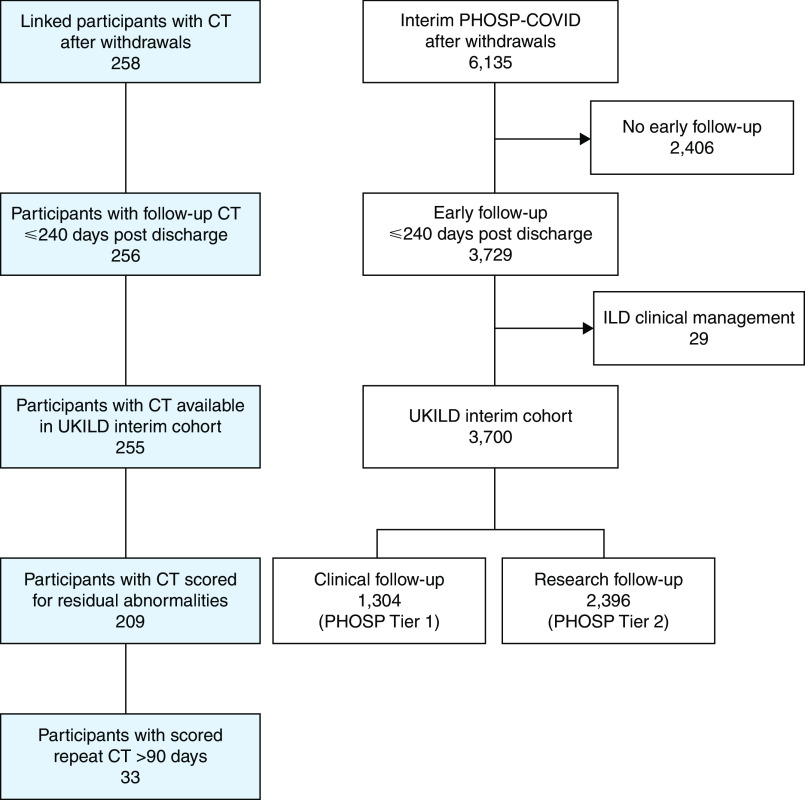
CONSORT flow diagram of UKILD post–COVID-19 study interim cohort definition. White boxes derived from the PHOSP-COVID database. Blue boxes represent computed tomography samples linked with Post-Hospitalization COVID (PHOSP-COVID) identifiers in a radiological database. CONSORT = Consolidated Standards of Reporting Trials; CT = computed tomography; ILD = interstitial lung disease; UKILD = UK interstitial lung disease consortium post–COVID-19 study.

A total of 3,700 PHOSP–COVID-19 participants reached the criteria for inclusion in the interim UKILD post–COVID-19 study cohort. This included 1,304 patients with data available through routine clinical care (Tier 1) and 2,396 who had completed an early follow-up research visit within 240 days of discharge (Tier 2) (Figure 1). We observed that 255 of 3,700 (6.9%) participants in the interim cohort had a linkable thoracic CT scan performed, 220 of 2,396 (9.2%) Tier 2 participants had CT scans performed, and 35 of 1,304 (2.7%) of Tier 1 participants had CT scans performed (*P* < 0.001). Of 255 participants with linked CT scans within 240 days of discharge (median, 113 days; interquartile range [IQR], 69–166) (Figure E1 in the online supplement), a total of 209 (82.0%) were visually scored with interrater agreement on 70% of scans (Cohen’s κ, 0.33). Participants with a CT scored were majority male (68.4%), White (68.9%), had a median age of 58 (52–67), and had a median time to early follow-up visit of 140 days (IQR, 106–170) (Table 1).

**Table 1.** UKILD Post–COVID-19 Study Interim Cohort Demographics

	Interim		CT score		Tier 1		Tier 2		
	*n* = 3700	%	*n* = 209	%	*n* = 1304	%	*n* = 2396	%	χ^2^ *P* value
Sex									0.091
Male	2,247	60.7	143	68.4	768	58.9	1,479	61.7	—
Female	1,450	39.2	66	31.6	535	41.0	915	38.2	—
Age									0.027
⩾60	1,801	48.7	99	47.4	667	51.2	1,134	47.3	—
<60	1,895	51.2	110	52.6	636	48.8	1,259	52.5	—
Ethnicity									<0.001
White	2,804	75.8	144	68.9	1,015	77.8	1,789	74.7	—
Asian	467	12.6	40	19.1	144	11.0	323	13.5	—
Black	223	6.0	15	7.2	56	4.3	167	7.0	—
Other	131	3.5	6	2.9	31	2.4	100	4.2	—
Missing	75	2.0	—	—	58	4.4	17	0.7	—
IMD									0.031
1 (most)	867	23.4	38	18.2	326	25.0	541	22.6	—
2	817	22.1	40	19.1	268	20.6	549	22.9	—
3	666	18.0	41	19.6	251	19.2	415	17.3	—
4	659	17.8	38	18.2	241	18.5	418	17.4	—
5 (least)	667	18.0	50	23.9	210	16.1	457	19.1	—
Missing	24	0.6	—	—	8	0.6	16	0.7	—
BMI									0.491
<25	262	7.1	22	10.5	45	3.5	217	9.1	—
25–<30	612	16.5	59	28.2	84	6.4	528	22.0	—
30–<40	880	23.8	67	32.1	121	9.3	759	31.7	—
⩾40	230	6.2	12	5.7	30	2.3	200	8.3	—
Missing	1,716	46.4	49	23.4	1,024	78.5	692	28.9	—
WHO severity									0.826
No O_2_ (i)	624	16.9	35	16.7	223	17.1	401	16.7	—
Noninvasive O_2_ (ii)	1,567	42.4	77	36.8	557	42.7	1,010	42.2	—
CPAP (iii)	860	23.2	34	16.3	306	23.5	554	23.1	—
IMV (iv)	645	17.4	63	30.1	217	16.6	428	17.9	—
CXR at follow-up									<0.004
Normal	1,289	34.8	70	33.5	511	39.2	778	32.5	—
Other	325	8.8	19	9.1	140	10.7	185	7.7	—
Abnormal	162	4.4	21	10.0	45	3.5	117	4.9	—
Missing	2,139	57.8	36	41.4	677	52.2	1,462	60.8	—
CT at follow-up									—
Linked records	255	6.9	209	100.0	35	2.7	220	9.2	<0.001
Scored	209	5.6	209	100.0	29	2.2	180	7.5	<0.001
Symptoms at follow-up									0.636
Present (worsen)	850	23.0	74	35.4	21	1.6	829	34.6	—
Present (no change)	319	8.6	21	10.0	11	0.8	308	12.9	—
Not present/improved	359	9.7	24	11.5	9	0.7	350	14.6	—
Missing	2,172	58.7	90	43.1	1,263	96.9	909	37.9	—
ppFVC at follow-up, %									—
⩾80	786	21.2	53	25.4	—	—	773	32.3	—
<80	297	8.0	29	13.9	—	—	294	12.3	—
Missing	2,617	70.7	127	60.8	1,288	98.8	1,329	55.5	—
ppDl_CO_ at follow-up, %									—
⩾80	333	9.0	37	17.7	—	—	333	13.9	—
<80	177	4.8	25	12.0	—	—	175	7.3	—
Missing	3,190	86.2	147	70.3	1,302	99.8	1,888	78.8	—
	Median	IQR	Median	IQR	Median	IQR	Median	IQR	
Age, yr	59	(50–68)	58	(52–67)	60	(51–70)	59	(50–67)	—
ppFVC	90.3	(78.6–101.7)	87.0	(75.0–98.8)	—	—	90.2	(78.6–101.6)	—
ppDl_CO_	87.6	(74.2–101.3)	84.7	(69.9–96.2)	—	—	87.5	(74.0–101.3)	—
Time to follow-up, d	127	(91–173)	140	(106–170)	101	(82–138)	141	(100–180)	—

*Definition of abbreviations*: BMI = body mass index; CT = computed tomography–chest; CXR = chest X-ray; IMD = index of multiple deprivation in quintiles; IQR = interquartile range; ppDl_CO_ = percent predicted Dl_CO_; ppFVC = percent predicted FVC; Symptoms = Patient Symptom Questionnaire breathless or cough; WHO = modified World Health Organization severity score.

Small numbers of less than 5 have been suppressed. χ^2^ performed between Tier 1 and Tier 2 on nonmissing categories.

**Figure 2.**
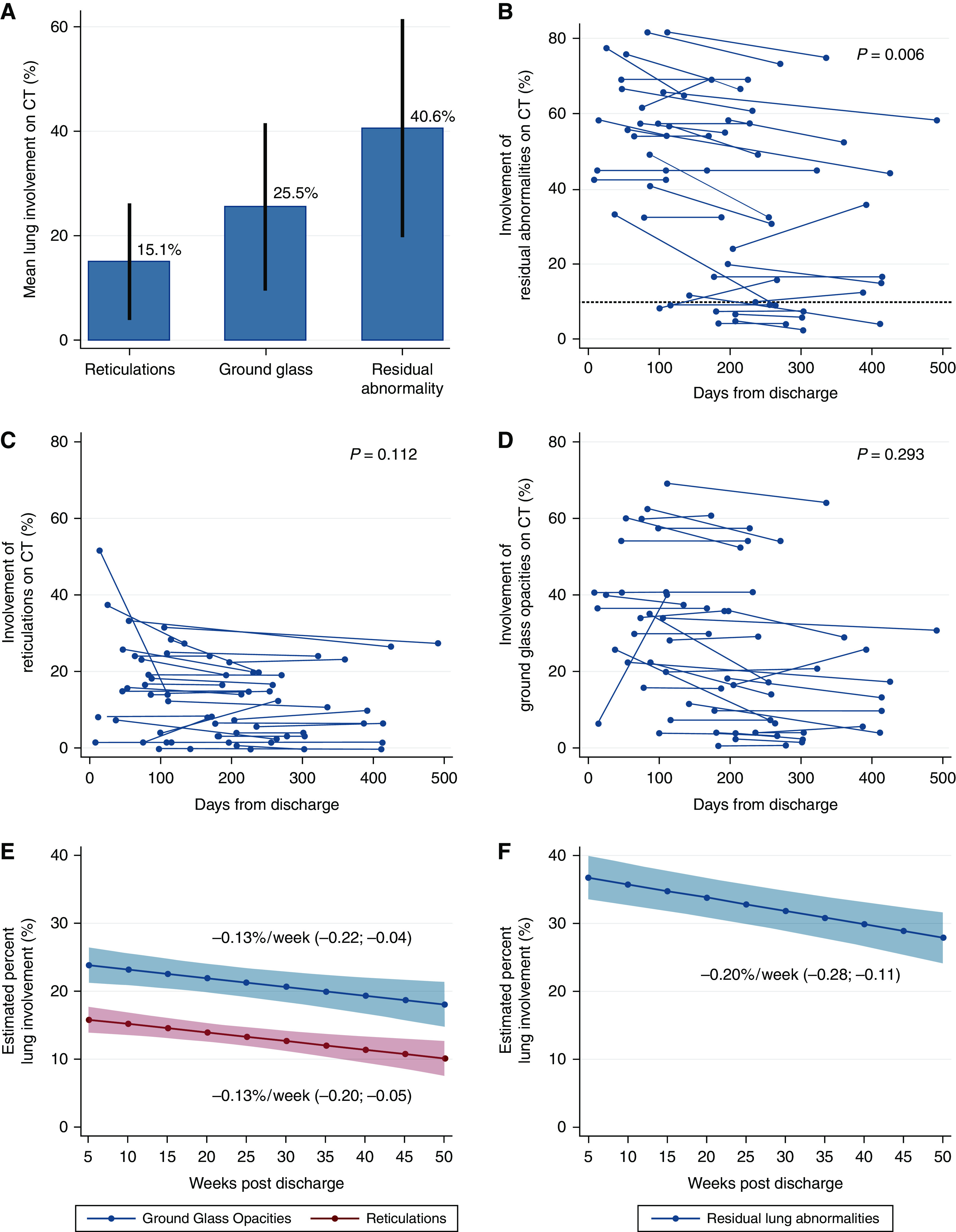
Extent of residual lung abnormalities on linked computed tomography. (*A*) Mean percentage lung involvement of reticulations, ground-glass opacities, and residual abnormalities within 240 days of discharge with visually scored involvement greater than 10%, presented with standard deviation (*n* = 166). Percentage lung involvement of (*B*) residual abnormalities, (*C*) reticulations, and (*D*) ground-glass opacities at initial and repeat computed tomography scans with greater than 90 days between (*n* = 33), with *P* values from paired *t* test. (*E*) Estimated percent lung involvement of ground-glass opacities (top, blue) and reticulations (bottom, red) from linear mixed effects by weeks after discharge (*n* = 209, scans = 242). (*F*) Estimated percent lung involvement of residual abnormalities from linear mixed effects by weeks after discharge, presented with mean weekly effect and 95% confidence intervals (*n* = 209, scans = 242). CT = computed tomography.

**Figure 3.**
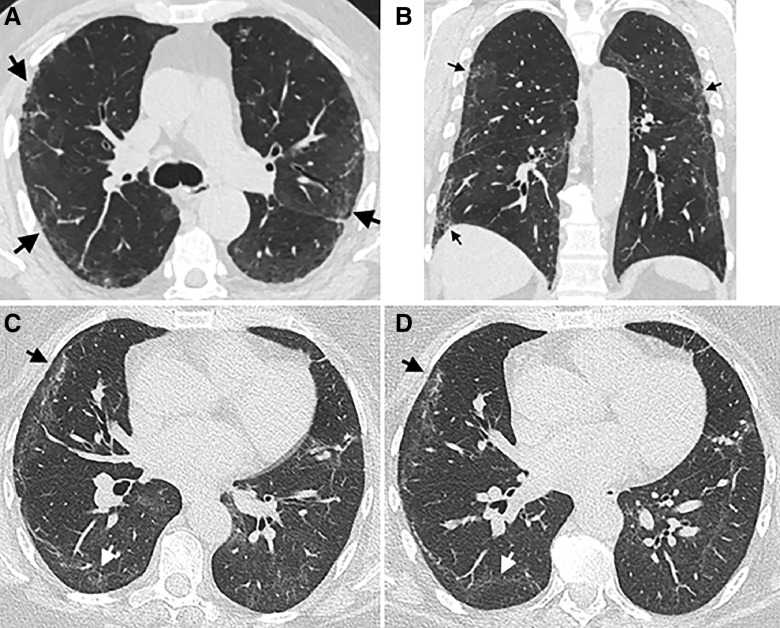
Representative computed tomography (CT) images of residual lung abnormalities. Representative (*A*) coronal and (*B*) axial noncontrast CT imaging from the same individual performed 137 days after discharge after a coronavirus disease (COVID-19) admission scored with 52.5% total lung involvement of residual lung abnormality, of which 18.3% was reticulation and 34.2% ground-glass opacity. Peripheral reticulation (arrows) is evident, surrounded by faint areas of ground-glass opacity. Representative coronal CT images from the same individual at (*C*) 114 days after discharge scored 56.8% lung involvement (28.5% reticulation and 28.3% ground-glass opacity), and (*D*) 239 days after discharge scored 49.2% total lung involvement (20.0% reticulation and 29.2% ground-glass opacity). Peripheral areas of reticulation (black arrow) and ground-glass opacity (white arrow) in the right lung.

Residual lung abnormalities greater than 10% were observed in 166 of 209 (79.4%) participants. Visual scoring of involvement indicated ground-glass opacities affected a mean of 25.5 ± 5.9% of the lung, reticulation a mean of 15.1 ± 11.0%, with residual abnormalities involved a mean of 40.6 ± 20.8% of the lung (Figure 2A). A total of 33 people had a repeat CT visually scored after a minimum of 90 days (median, 161 d; IQR, 109–187), 28 of 33 (84.8%) of whom were classified with residual abnormalities greater than 10% on the initial scan, with 26 of 28 (92.9%) observed to have greater than 10% involvement in subsequent scans. In paired analysis, the overall change in residual lung abnormalities was −3.62% (95% confidence interval [CI], −6.10 to −1.13; *P* = 0.006) (Figure 2B). The involvement of lung reticulations and ground-glass opacities did not significantly change with a mean difference of −2.08% (95% CI, −4.66 to 0.51; *P* = 0.112) and −1.54% (95% CI, −4.74 to 1.39; *P* = 0.293), respectively (Figures 2C and 2D). Using all scored CT scans, the mean weekly change in lung involvement was estimated at −0.13% per week (95% CI, −0.20 to −0.05) for reticulations and −0.13% per week (95% CI, −0.22 to −0.04) for ground-glass opacities (Figure 2E). The weekly change in residual lung abnormalities was −0.20% per week (95% CI, −0.28 to −0.11) (Figure 2F). Representative CT images of residual lung abnormality demonstrated persistent involvement more than 100 days after discharge (Figure 3).

Overall, the median time to follow-up in the UKILD interim cohort (*N* = 3,700) was 127 days (IQR, 91–173), the median age was 59 (IQR, 50–68), and the cohort was majority male (60.7%). Tier 1 participants (*n* = 1,304) had a median time to follow-up of 101 days (IQR, 82–138), a median age of 60 (IQR, 51–70), and the majority were male (58.9%); demographics were similar in Tier 2 participants (*n* = 2,396) with a median time to research visit of 141 days (IQR, 100–180), a median age of 59 (IQR, 50–67), and the majority male (61.7%) (Table 1). There was minimal evidence of systematic bias in the characteristics between Tier 2 and Tier 1 participants in nonmissing data (Table 1), although the representation of people aged below 60 was greater in Tier 2 participants (52.5% vs. 48.8%; *P* = 0.027); similarly, there were small differences in the representation of ethnicity (*P* < 0.001), greater representation of the lowest deprivation quintile (19.1% vs. 16.1%; *P* = 0.031), as well as lower representation of normal CXR (32.5% vs. 39.2%; *P* = 0.004).

Tier 2 participants had a median ppFVC at research follow-up of 90.2% (IQR, 78.6–101.6) with missing records at 55.5%, whereas median ppDl_CO_ was 87.5% (IQR, 74.0–101.3) with missing records at 78.8%; lung function was largely missing in the routine follow-up of Tier 1 participants. We observed 34.6% of people reported worsening cough or dyspnea since discharge in Tier 2. ILD diagnostic criteria of lung function (ppFVC and ppDl_CO_), CXR, and symptoms were frequently missing, particularly in Tier 1 of clinical follow-up (Figure E2). In Tier 1, 578 of 1,304 (44.3%) participants were missing data on all four characteristics at interim analysis, whereas in Tier 2, 362 of 2,396 (15.1%) participants were missing data on all four characteristics. In contrast, a total of 202 (8.4%) Tier 2 participants had complete data on all, and no Tier 1 participants had complete lung function, CXR, or symptom data. In the subsample of participants with scored CTs, data was missing at a rate similar to Tier 2 for lung function (ppDl_CO_, 70.3% and ppFVC, 60.8%), CXR (47.4%), and Patient Symptom Questionnaire (43.1%) (Table 1).

### Risk of Residual Lung Abnormalities and Persistence Over Time

Univariate risk ratios were calculated to assess the risk of residual lung abnormalities greater than 10% on CT. A greater risk was observed in males (risk ratio [RR], 1.42; 95% CrI, 1.17–1.77) and in those over 60 years of age (RR, 1.22; 95% CrI, 1.06–1.40). Clinical indicators, including severe illness on admission requiring CPAP, IMV, or ECMO (RR, 1.40; 95% CrI, 1.23–1.63), abnormal CXR findings (RR, 1.40; 95% CrI, 1.22–1.61), and ppDl_CO_ less than 80% (RR, 1.26; 95% CrI, 1.02–1.58) were also associated with greater risk, with consistent effects for the relative mean difference of percent involvement after adjustment for sex and deprivation quintile (Table 2).

**Table 2.** Risk Factors of Residual Lung Abnormalities on Computed Tomography

Characteristic	Risk Factor Present, %	Risk Factor Absent, %	Univariate Risk Ratio	95% Credible Interval	Estimated Mean Difference, %	95% Credible Interval	Adjusted Mean Difference, %	95% Credible Interval
Male	87.4	62.1	1.42	(1.17 to 1.77)	12.46	(5.76 to 19.59)	11.26	(4.24 to 18.04)
Age ⩾60, yr	87.9	71.8	1.22	(1.06 to 1.40)	8.29	(2.11 to 14.44)	8.57	(3.61 to 6.16)
Non-White	78.5	79.9	0.97	(0.84 to 1.12)	3.48	(−3.78 to 10.88)	3.84	(−4.95 to 9.37)
IMD (Q1 and 2)	87.2	74.4	1.17	(1.02 to 1.34)	6.91	(0.38 to 13.33)	6.28	(−0.31 to 12.91)
BMI >30	87.3	71.6	1.22	(1.04 to 1.45)	3.93	(−3.70 to 11.52)	4.54	(−2.40 to 11.65)
CPAP/IMV	93.8	67.0	1.40	(1.23 to 1.63)	20.56	(14.80 to 26.36)	20.14	(14.34 to 25.69)
aCXR	100.0	73.0	1.40	(1.22 to 1.61)	14.96	(3.89 to 25.78)	11.54	(0.53 to 21.59)
ppFVC <80	86.2	79.3	1.07	(0.85 to 1.31)	10.40	(−0.90 to 22.00)	11.99	(−0.14 to 23.52)
ppDl_CO_ <80	96.0	75.7	1.26	(1.02 to 1.58)	19.04	(7.65 to 30.71)	15.31	(2.84 to 28.06)
PSQ worse	78.4	80.0	0.99	(0.81 to 1.21)	4.49	(−4.58 to 13.54)	4.71	(−4.31 to 13.87)

*Definition of abbreviations*: aCXR = abnormal chest X-ray; BMI = body mass index; CPAP/IMV = continuous positive airway pressure or invasive mechanical ventilation; IMD = Index of Multiple Deprivation; Q = quintile; ppDl_CO_ = percent predicted Dl_CO_; ppFVC = percent predicted FVC; PSQ = Patient Symptom Questionnaire.

Percentage of nonmissing case observations reaching greater than 10% threshold of residual lung abnormalities according to risk factor being present or absent. Univariate risk ratio of greater than 10% threshold of residual lung abnormalities and 95% credible interval derived from binomial regression, mean effect difference in the percentage lung involvement in which risk factor present relative to risk factor absent estimated from univariate linear regression and adjusted for sex and index of multiple deprivation.

Three significant clinical indicators were selected to index the risk of residual lung abnormalities after COVID-19 in the remaining cohort (*n* = 3,491) on the basis of combined thresholds: ppDl_CO_ less than 80%; abnormal CXR; and severe illness on admission. Individuals were considered to be at very high risk when reaching the defined thresholds in all three indicators (risk index four), high risk when two thresholds were reached (risk index three), or moderate risk if reaching ppDl_CO_ or CXR thresholds alone (risk index two). Individuals reaching the threshold of the severity of illness on admission alone were considered low risk in the absence of other indicators (risk index one). Those who did not reach any threshold were considered very low risk (risk index zero). A total of 14 of 3,419 (0.4%) participants were considered very high risk, 143 of 3,419 (4.1%) high risk, 116 of 3,419 (3.3%) moderate risk, 1,256 of 3,419 (36.0%) low risk, and 1,962 of 3,419 (56.2%) very low risk (Table 3). Combined, 273 of 3,419 (7.8%) participants in strata of moderate to very high risk were defined as at-risk, and 8 of 46 (17.4%) participants with an unscored clinically indicated CT were at-risk. In sensitivity analyses applying risk stratification to Tier 2 alone, 231 of 2,219 (10.4%) participants were at moderate to very high risk, including 20% of those with an unscored clinically indicated CT (Table 3).

**Table 3.** Risk Stratification of Residual Lung Abnormalities in Unscored UKILD Post–COVID-19 Study Interim Cohort

Interim Cohort				
Strata	Unscored (*n* = 3,491), *n*	%	Sensitivity (*n* = 2,219), *n*	%
Very high	14	0.4	14	0.6
High	143	4.1	123	5.5
Moderate	116	3.3	94	4.2
Low	1,256	36.0	767	34.6
Very low	1,962	56.2	1,221	55.0

Linked CT: Unscored				
	Interim (*n* = 46), *n*	%	Sensitivity (*n* = 40), *n*	%
At-risk	8	17.4	8	20.0
Low risk	38	82.6	32	80.0

*Definition of abbreviation*: CT = computed tomography.

Risk strata: Very high (all three risk factors present [abnormal chest X-ray, percent predicted Dl_CO_ less than 80%, and severe admission requiring continuous positive airway pressure or invasive mechanical ventilation]); High (at least two risk factors present); Moderate (either abnormal chest X-ray or percent predicted Dl_CO_ less than 80% present); Low (severe admission present only); Very low (risk factors not present). Missing data were imputed at the reference category. Percent denominator is the interim cohort without linked, scored computed tomography (*n* = 3,491) and sensitivity analysis within Tier 2 research visit participants (*n* = 2,219). Moderate to very high risk combined with at-risk and low to very low risk combined with low risk quantified in people with unscored linked computed tomography.

No differences were observed between at-risk participants (*n* = 273) and participants with greater than 10% residual abnormalities on CT (*n* = 166) according to a representation of males, older age, ethnicity, deprivation, body mass index, severity of admission, ppFVC less than 80%, or Patient Symptom Questionnaire (Table E1). There was a lower representation of normal CXR in the at-risk group (14.7% vs. 30.1%; *P* < 0.001) and more representation of ppDl_CO_ less than 80% (55.3% vs. 14.5%; *P* < 0.001). The percentage of people who did not have a severe admission requiring CPAP, ECMO, or IMV was similar in both groups (44.3% vs. 45.2%), whereas CXR was missing in 26.0% of the at-risk group and 48.2% of people with residual abnormalities scored.

Comparing at-risk participants to low-risk participants, there were more records of immunosuppressant (18.3% vs. 9.9%; *P* = 0.001) and corticosteroid treatment (35.3% vs. 26.5%; *P* = 0.019) preadmission, intensive care unit stays (50.0% vs. 33.4%; *P* < 0.001), and complications of acute respiratory distress syndrome (ARDS; 25.0% vs. 13.7%; *P* < 0.001) (Table E2). In addition, there were more recorded unscheduled emergency visits after discharge (34.8% vs. 25.2%; *P* = 0.001), with a greater representation of visits in which patients presented with symptoms of shortness of breath (33.7% vs. 24.3%; *P* = 0.046). Findings were similar in comparisons of CT-scored residual lung abnormalities greater than 10% compared with those not reaching this threshold, although statistical significance was not always met (Table E2).

On the basis of the distribution of at-risk cases, the prevalence of residual lung abnormalities after COVID-19 hospitalization was estimated at 8.51% (95% CrI, 7.56–9.51) using noninformative priors, or 6.49% (95% CrI, 5.75–7.27) with skeptical priors on the basis of ILD population prevalence estimated at 1 in 1,000 (Table 4 and Figure E3) (18, 19). In sensitivity analyses on the basis of Tier 2 distribution, the prevalence of residual lung abnormalities after COVID-19 hospitalization was estimated at 11.67% (95% CrI, 10.28–13.14) using noninformative priors, or 7.74% (95% CrI, 6.79–8.72) using skeptical priors.

**Table 4.** Prevalence Estimate of Residual Lung Abnormalities Greater than 10% After COVID-19 Hospitalization

Model	Prevalence, %	95% CrI	Prior	a	b	DIC
1	8.51	(7.56–9.51)	Uniform	1	1	9.38
1-i	8.48	(7.52–9.49)	Jeffreys	0.5	0.5	9.45
1-ii	6.49	(5.75–7.27)	Skeptical	1	1,000	28.67
1-iii	7.37	(6.53–8.24)	Power	1	1,000	14.99
2	11.67	(10.28–13.14)	Uniform	1	1	9.20
2-i	11.61	(10.19–13.04)	Jeffreys	0.5	0.5	9.27
2-ii	7.74	(6.79–8.72)	Skeptical	1	1,000	45.97
2-iii	9.32	(8.17–10.54)	Power	1	1,000	20.91

*Definition of abbreviations*: Crl = credible interval; DIC = deviance information criterion.

The estimated prevalence of greater than 10% residual lung abnormalities on computed tomography after hospitalization for coronavirus disease (COVID-19), derived from posterior mean and 95% credibility interval using binomial distributions of at-risk versus low-risk numbers in interim UK Interstitial Lung Disease Consortium (UKILD) post–COVID-19 study cohort. Model 1, overall; Model 2, Tier 2 research visit participants. Uniform priors and sensitivity analysis with Jeffreys noninformative (i), skeptical informative priors (ii), and skeptical informative priors with power at 50% weighting (iii). β prior distributions defined using cases (a) and noncases (b). DIC presented to interpret the model.

## Discussion

These data demonstrate that residual lung abnormalities were visually identifiable on clinically indicated thoracic follow-up CT imaging in a substantial proportion of patients within 8 months of discharge after COVID-19 hospitalization. The involvement of scored residual lung abnormalities minimally declined per week after discharge, and minimal resolution was observed in paired subsequent scans at least 90 days apart. Key clinical risk factors associated with residual abnormalities in the early follow-up period included abnormal CXR, ppDl_CO_ less than 80%, and severe admissions requiring invasive support (IMV, CPAP, or ECMO). In those without a scored CT, 0.4% were in very-high–risk strata (all three indicators present), 4.1% in high-risk strata (any two indicators present), and 3.3% in moderate-risk strata (presence of either ppDl_CO_ less than 80% or abnormal CXR, alone). Combining these risk strata, 7.8% of the interim cohort had suspected residual lung abnormalities after COVID-19 hospitalization, which increased to 10.4% in sensitivity analysis on those with planned research follow-up. On the basis of Bayesian modeling, we estimate the prevalence of suspected residual lung abnormalities with greater than 10% lung involvement to be up to 11.7% in people hospitalized with acute COVID-19 infections before March 2021.

This UKILD Post–COVID-19 Study interim analysis of residual abnormalities in patients hospitalized for COVID-19 offers the largest assessment of prevalence in hospitalized individuals to date and is consistent with findings from a number of smaller studies that demonstrate persistent radiological patterns and impaired gas transfer during extended follow-up of patients with COVID-19 (20–23). At the time of this interim analysis, it is not possible to determine whether the observed residual lung abnormalities represent early ILD with potential for progression or whether they reflect residual pneumonitis that may be stable or resolve over time (24). The 10% threshold used was determined to support the distinction of interstitial lung damage from interstitial lung abnormalities (15). Longer-term follow-up and mechanistic studies will be required to determine the clinical trajectory of these observations.

When linked longitudinal scans were available, most patients did not show evidence of substantial improvement, although such clinically requested CTs may be overrepresented by those with slower recovery. However, approximately half the people with visually scored residual abnormalities above the 10% threshold did not require CPAP, IMV, or ECMO during their admission and less than one quarter had ARDS recorded as a complication, suggesting medium- and longer-term disability consequent to severe COVID-19 infection, consistent with prior studies (18).

The risk factors for a residual abnormality scored in the CT subsample (abnormal CXR, ppDl_CO_ less than 80%, and severe admissions requiring invasive support) were applied to the remaining hospitalized cohort to generate clinically applicable risk strata. For participants in receipt of a clinically indicated but unscored CT, 17.4% of people were in moderate- to very-high–risk strata for residual lung abnormalities (sensitivity, 20.0%). These rates were similar to meta-analysis estimates of the percentage of clinically indicated CT scans with radiological patterns suggestive of fibrosis (29%; 95% CI, 22–37%) and people with impaired gas transfer (17%; 95% CI, 13–23), neither of which were associated with the timing of follow-up within the first year after COVID-19 (25). In paired CT scans greater than 90 days apart, we demonstrate no significant difference in the mean change for percent involvement of reticulations and ground-glass opacities, whereas the scored involvement of reticulations and ground-glass opacities on the basis of all CT scans declined by 0.13% per week of study from discharge, suggesting persistence over time in at-risk groups.

Differences between individuals at moderate to very high risk and those at lower risk suggested more immunosuppressant and corticosteroid treatment preadmission, ICU stays, and ARDS complications, as well as further unscheduled emergency visits after discharge both overall and including a presentation with breathlessness. Classification of at-risk participants using clinically applicable strata identified those who may have had a more severe viral injury and inflammatory response during acute infection, as well as subsequent respiratory exacerbations after COVID-19. Recent analysis identified a hyperinflammatory phenotype of COVID-19–related ARDS was associated with worse outcomes, with better survival linked to corticosteroid treatment (26). Surviving a hyperinflammatory response to COVID-19 may be consistent with residual lung abnormalities, including fibrosing nonspecific interstitial pneumonia and alveolar damage (27).

Residual lung abnormalities after COVID-19 were not uncommon in this hospitalized population and may persist long-term, but indicators that could support diagnosis and clinical management of lung disease were frequently unavailable. Considering approximately 280,000 people were discharged after confirmed COVID-19 admission in the United Kingdom National Health Service by the end of March 2021 (28), these results emphasize the importance for health services to undertake active radiological and physiological monitoring, especially in people at moderate or above risk (15).

### Strengths and Limitations

The UKILD long–COVID-19 Study interim cohort excluded participants with any evidence of ILD before hospitalization, and we used informative skeptical priors and power priors for more conservative estimates of prevalence, which continued to suggest a substantial burden of residual lung abnormalities after COVID-19 hospitalization. The approach we report can be reasonably applied to other cohorts and time points, with current findings used as informative priors for updating Bayesian inference.

Although included CTs were assumed to be representative of clinically indicated radiology, this is limited by local management protocols, the timing of services, and changes to healthcare service prioritization during the COVID-19 pandemic, which increases the chances of selection and ascertainment bias. Furthermore, individuals with linked CT may have unrecorded preexisting disease or present with radiological patterns other than reticulation and ground-glass opacities. Fair interrater agreement (κ, 0.33) of CT scoring was observed, representing agreement in 70% of scans.

We recognize these interim findings may also be limited by misclassification. Descriptive analyses identified substantial missing data in clinical risk factors, limiting multiple imputation techniques. We used dichotomized thresholds with single data imputation at the reference category to support risk strata classification, maintain denominators, and provide conservative estimates. In contrast, lung involvement of reticulation and ground-glass opacities was frequently scored on CTs that were clinically indicated, contributing to selection bias. It is similarly likely that repeat CT scans reflect a sample of individuals that did not experience clinical improvement over time. We report estimates from multilevel models to support the interpretation of residual lung abnormalities over time.

Although our findings are on the basis of people hospitalized with mixed severity of COVID-19 infection, we recognize that they may not be generalizable to all populations, especially those not admitted to the hospital. Severe admissions requiring CPAP or IMV were overrepresented in the PHOSP–COVID-19 dataset relative to hospitalized survivors of COVID-19 (14). Linked clinical admission data suggested 50% of at-risk individuals and those scored with residual abnormalities attended ICUs during admission, and up to 25% had complications of anemia and ARDS. Furthermore, these data reflect people who were discharged before the end of March 2021 and do not represent later severe acute respiratory syndrome coronavirus 2 (SARS-CoV-2) variants in fully vaccinated populations that more frequently led to milder infections.

### Conclusions

Thresholds of ppDl_CO_, CXR, and severity of admission can stratify the risk of residual abnormalities on CT involving more than 10% of the lung, informing clinical management, particularly of individuals meeting moderate- to very-high–risk strata. Longitudinal analysis of CT scans suggested the persistence of abnormalities over study time, although the longer-term functional consequence is unknown and may be limited by clinical indication. These findings highlight the importance of radiological and physiological monitoring of patients at both early and later follow-ups and suggest up to 11% of people discharged from an acute COVID-19 admission are at risk of residual lung abnormalities. Further study is required to elucidate the progressive development of radiological patterning or resolution over time.
